# Hispanic health in the USA: a scoping review of the literature

**DOI:** 10.1186/s40985-016-0043-2

**Published:** 2016-12-07

**Authors:** Eduardo Velasco-Mondragon, Angela Jimenez, Anna G. Palladino-Davis, Dawn Davis, Jose A. Escamilla-Cejudo

**Affiliations:** 1 College of Osteopathic Medicine, Touro University California, 1310 Johnson Lane; H-82, Rm. 213, Vallejo, CA 94592 USA; 2 Fay W. Boozman College of Public Health, University of Arkansas for Medical Sciences, Little Rock, USA; 3 Bezley Institute for Health Law and Policy, Loyola University Chicago, Chicago, USA; 4 St. Louis University School of Medicine, St. Louis, USA; 5 Regional Advisor on Health Information and Analysis, Pan American Health Organization/World Health Organization, Foggy Bottom, USA

**Keywords:** Hispanics, Latinos, Scoping study, Social determinants of health, Health care inequalities, Health care access

## Abstract

Hispanics are the largest minority group in the USA. They contribute to the economy, cultural diversity, and health of the nation. Assessing their health status and health needs is key to inform health policy formulation and program implementation. To this end, we conducted a scoping review of the literature and national statistics on Hispanic health in the USA using a modified social-ecological framework that includes social determinants of health, health disparities, risk factors, and health services, as they shape the leading causes of morbidity and mortality. These social, environmental, and biological forces have modified the epidemiologic profile of Hispanics in the USA, with cancer being the leading cause of mortality, followed by cardiovascular diseases and unintentional injuries. Implementation of the Affordable Care Act has resulted in improved access to health services for Hispanics, but challenges remain due to limited cultural sensitivity, health literacy, and a shortage of Hispanic health care providers. Acculturation barriers and underinsured or uninsured status remain as major obstacles to health care access. Advantageous health outcomes from the “Hispanic Mortality Paradox” and the “Latina Birth Outcomes Paradox” persist, but health gains may be offset in the future by increasing rates of obesity and diabetes. Recommendations focus on the adoption of the Health in All Policies framework, expanding access to health care, developing cultural sensitivity in the health care workforce, and generating and disseminating research findings on Hispanic health.

**Electronic supplementary material:**

The online version of this article (doi:10.1186/s40985-016-0043-2) contains supplementary material, which is available to authorized users.

## Background

Hispanics are the largest ethnic minority in the USA; in 2014, Hispanics comprised 17.4% of the US population (55.4 million), and this percentage is expected to increase to 28.6% (119 million) by 2060. Hispanics in the USA include native-born and foreign-born individuals immigrating from Latin America, the Caribbean, and Spain [1].

Hispanics are disproportionately affected by poor conditions of daily life, shaped by structural and social position factors (such as macroeconomics, cultural values, income, education, occupation, and social support systems, including health services), known as social determinants of health (SDH). SDH exert health effects on individuals through allostatic load [2], a phenomenon purported to cause chronic stress, which elicits behavioral risk factors such as poor diet, sedentary behaviors, and substance use, as well as biological processes such as circadian rhythm disruption, cytokine responses, and inflammation [3].

SDH are implicated in health inequalities, which are defined as health conditions that typically affect disproportionally certain socioeconomic, ethnic, and gender population subgroups [4]. Health inequalities particular to Hispanics are also those related to their socioeconomic status, cultural background, employment, and foreign-born or undocumented status [5, 6]. Hispanics residing in the USA are on average 15 years younger, four times more likely to not have finished high school, twice as likely to live below the poverty line, and 20 times less likely to speak proficient English than non-Hispanic Whites (NHW) [1, 7]. Hispanic women are also a growing demographic group that endure adverse social and health conditions and lack of access to health care [8].

Risk factors for non-communicable diseases (NCDs), coupled with decreased health care access make Hispanics disproportionately vulnerable to disease and death. Hispanics endure major health risks such as obesity, teen pregnancy, and tobacco use, among others. Significant differences in risk factors, morbidity, mortality, and access to health care can also be observed among Hispanics by country of origin [4, 9, 10]. The most recent reports show that the leading causes of disease among Hispanics are heart disease, cancer, and high blood pressure, while the leading causes of death are cancer, heart disease, and unintentional injuries.

Health care services in the USA are provided mainly through employer-based health insurance, Medicare, and Medicaid. Employer-based insurance is usually privately purchased. Medicare insures people 65 years and older (or younger than 65 with disabilities), and Medicaid is a social welfare program for low-income population. In 2010, President Obama signed into law the Patient Protection and Affordable Care Act (ACA) to expand health care protection by increasing insurance coverage, expanding Medicaid, decreasing health care costs, allowing provider choice and improving the quality of care [11]. Historically, Hispanics in the USA have less access to health services and they utilize fewer preventive care services than other ethnic groups, with 30% reporting no health insurance before the implementation of the ACA in 2014, compared to 11% for NHWs [12].

Several literature reviews on Hispanic and Latino/Latina Health have been conducted in the past; most consist of cross-sectional or qualitative studies focusing separately on acculturation, health disparities, risk behaviors, specific health conditions, and access to health care. Some of them focus on specific age and gender groups or on country of origin, migrant workers, and undocumented populations [13–21], while some others discuss the Latina Birth Outcomes and Hispanic Mortality Paradoxes [22, 23].

After a preliminary review of the literature on the topic [7, 11, 24], we identified a lack of a unified framework to assess Hispanic health in the USA, as well as the need to conduct a scoping review of the literature on the main Hispanic health needs and health policies and services—including the Latina Birth Outcomes Paradox and the Hispanic Mortality Paradox—to help inform policy- and decision-making for improving Hispanic health in the USA. Such is the objective of this review paper.

### Conceptual framework

**Fig. 1:**
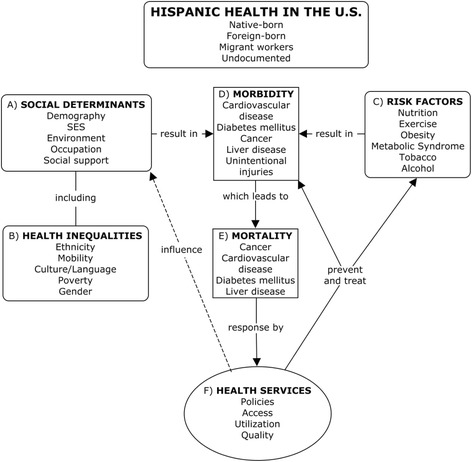
A framework to analyze Hispanic health in the USA

After discussing several conceptual frameworks, and to accomplish our objective, we developed a modified conceptual framework based on the social-ecological model [25] and the lifespan biopsychosocial model [26]. This comprehensive framework embodies the complex interactions—with synergistic and antagonistic effects—between social, biological, and psychological constructs of health (Fig. 1). It posits that distal variables pertaining to SDH (Fig. 1, A)—some of which include the main variables of health inequalities (Fig. 1, B)—operate as stressors that elicit epigenetic, biological, and psychological effects on individuals, resulting in health, disease (Fig. 1, D), and death (Fig. 1, E). They also interact with proximal variables such as risk factors (Fig. 1, C) (diet, obesity, physical inactivity, smoking, alcohol), in the causal pathways leading to health and disease.

Components of the framework do not have unidirectional cause-effect temporality but rather compose causality networks and trajectories that influence each other over time in interconnected, multi-directional cycles. For example, social support systems (education, labor, sports, food programs, recreation) include health services that serve as determinants of health which influence health needs and risks; however, changes in health needs and risks in turn modulate health services (Fig. 1, F), which—through prevention, treatment, and rehabilitation services—have an effect on health needs and risks. These causal networks exert more nuanced effects across Hispanic population subgroups by country of origin, foreign-born status, and migrant and undocumented status.

The wide-ranging breadth of this framework would be best approached through a comprehensive review and detailed analysis that would be too extensive for this review paper. Thus, we limit our review and analyses to the main Hispanic health priorities, as reported in the literature and based on the authors’ expert consensus.

## Methods

We utilized Arksey and O’Malley’s [27] scoping methodology to conduct our review. Scoping studies allow reviewing of both scientific and “gray” literature to answer broad research questions. They are useful to “map key concepts underpinning a research area and the main sources and types of evidence available.” Scoping studies also serve to identify research gaps and to summarize and disseminate research findings to stakeholders and policymakers who would otherwise have to obtain information from multiple sources [28].

**Fig. 2:**
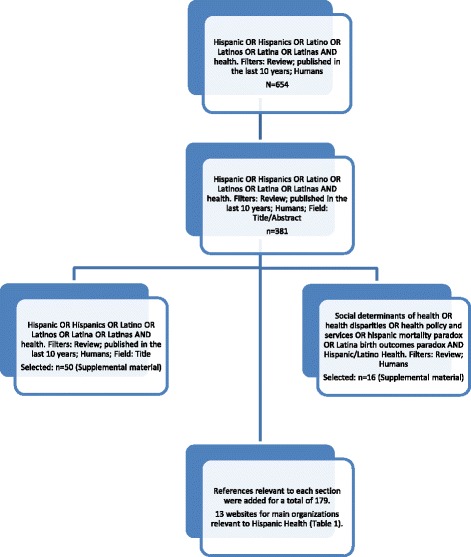
Literature review flowchart

We followed the five stages of a scoping study:

1. Identify the research question. Different from systematic reviews, where research questions are specific and focused on a particular type of study design, scoping studies seek to answer broader questions and collect data from different types of information sources. They also allow iterative rather than linear analytical processes to fine-tune the research focus in a way that the information is useful for decision-making and further research. The research question to pursue in this scoping review is, *What are the current priority issues, needs and services germane to the health of Hispanics in the USA?*
2. Identify relevant studies. Guided by our framework, we searched the literature for comprehensive Hispanic health review documents in electronic databases, government websites and agencies, and civil society organizations addressing Hispanic health. The first step was to find out whether there were any recent comprehensive reviews addressing our research question. Figure 2 shows a flowchart of our citation selection process. In PubMed, we used the Boolean search terms “*Hispanic OR Hispanics OR Latino OR Latinos OR Latina OR Latinas AND health*,” restricted to “*review*” and “*10 years*,” which yielded 654 citations, too many to review and too many tangential to our research question. After a cursory review of recent titles and abstracts, we added “*in title/abstract*” as a filter, which produced 381 citations. After eyeballing the references, there were still many tangential to our study questions. After a more targeted approach (Fig. 2), we selected 66 citations (as of September 2016) that were more specific to our research question (see Additional file 1). A thorough review of those citations supported our objective to conduct a scoping study using a unified framework of Hispanic health to answer our research question.
3. Study selection. A review of the initial reference list containing 66 citations on “Hispanic health reviews” showed that only one comprehensive review had been published in the previous 10 years [7], although it focused mostly on health needs and use of health services, not reflecting our more comprehensive conceptual framework and study design. While conducting this scoping review (alerted by peer-reviewers), a special issue on Latino Health was published [23], which seemed to supplant content in our review; however, the four papers in that issue refer to specific Hispanic health topics: cancer, cardiovascular disease, health promotion, and health issues in general, with no unifying framework. Upon completion of our literature search, a total of 366 references were included in an EndNote© database. For this paper, we selected only citations pertinent to each of the components of the conceptual framework of Hispanic health presented in Fig. 1, for a total of 179 citations (Fig. 2). All retrieved papers were made available online to the authors in a shared Dropbox file for online remote access.
4. Charting the data. A review of the first reference dataset containing 66 citations showed—with much overlap—that there were 26 review papers focusing mainly on social determinants of health and health disparities, 20 on health needs and risk factors, and 20 on health services (see Additional file 1) versing on diverse topics. We were able to retrieve 42 of the initial 66 reviews. All other references were added as authors reviewed and retrieved materials from different information sources (PubMed, Internet, books), for each component of the conceptual framework.
5. Collating, summarizing, and reporting results. We reviewed and selected papers, documents, and websites systematically to develop the sections on social determinants of health and health disparities, health risks, morbidity and mortality, health services, and the Hispanic and Latina paradoxes. Authors discussed and agreed upon references to be added for each section. Table 1 was prepared to show the main organizations addressing Hispanic health.

**Table 1:** Major Hispanic health agencies and initiatives in the USA

Organizations (*n* = 13)	Initiatives
Office of Minority Health	“…addresses disease prevention, health promotion, risk reduction, healthier lifestyle choices, use of health care services and barriers to health care for racial and ethnic minorities.” http://minorityhealth.hhs.gov/omh/browse.aspx?lvl=3&lvlid=64
CDC vital signs Hispanics	“…includes a Morbidity and Mortality Weekly Report Early Release, a graphic fact sheet and website, a media release, and social media tools. Most of the materials are available in English and Spanish.” http://www.cdc.gov/vitalsigns/hispanic-health/
US Office of Disease Prevention and Health Promotion	“…seeks to engage organizations, professionals, policymakers, communities, individuals, and families in a linked, multi-sector effort to improve health literacy” http://health.gov/communication/initiatives/health-literacy-action-plan.asp
Federal Government; US Centers for Medicare & Medicaid Services	“…health insurance exchange website operated under the United States federal government under the provisions of the Patient Protection and Affordable Care Act” https://www.cuidadodesalud.gov/es/
Study of Latinos	“…multi-center epidemiologic study in Hispanic/Latino populations to determine the role of acculturation in the prevalence and development of disease, and to identify risk factors playing a protective or harmful role in Hispanics/Latinos.” https://www2.cscc.unc.edu/hchs/view/biblio/year
Robert Wood Johnson Foundation Center for Health Policy	“…provides the most in-depth views to date on Latinos’ experiences with the Affordable Care Act (ACA) in the five years since its passage.” http://www.latinodecisions.com/files/1214/2707/3700/UNM_RWJF_Center_Toplines_Posted.pdf
League of United Latin American Citizens (LULAC)	“[this]…initiative is a comprehensive approach designed to reach Latinos across the United States and Puerto Rico to address health disparities in our communities.” http://lulac.org/programs/health/
National Hispanic Medical Association (NHMA)	“…to empower Hispanic physicians to lead efforts to improve the health of Hispanic and other underserved populations in collaboration with Hispanic state medical societies, residents, and medical students, and other public and private sector partners.” http://nhmamd.org/
National Council of La Raza	“We partner with Affiliates across the country to serve millions of Latinos in the areas of civic engagement, civil rights and immigration, education, workforce and the economy, health, and housing.” www.nclr.org
United States - Mexico Border Health Commission	“…addresses border health challenges by advancing initiatives that improve the health status of border residents.” http://www.borderhealth.org/
Migrant Clinicians Network	“We bring education, technical assistance, peer support, and advocacy to the field, creating a chain of connection and commitment that makes everyone stronger and more effective as we unite for one cause: health justice for the mobile poor.” http://www.migrantclinician.org/
National Alliance for Hispanic Health.	“We work to insure that health incorporates the best of science, culture, and community” http://www.hispanichealth.org/
Rand Corporation Center for Latin American Social Policy	“…dedicated to improving the well-being of the Latin American population and conducts objective, independent research on topics relevant to Latin Americans living and working at home and in the United States.” http://www.rand.org/labor/centers/clasp.html

We did not conduct the optional sixth stage of a scoping study: “Consultation.”

The following sections present the main components of Hispanic health, as outlined in our conceptual framework: social determinants of health and health inequalities, health risks, morbidity and mortality, health services and the Latina Birth Outcomes and Hispanic Mortality Paradoxes. Special subpopulations are emphasized where information was deemed important.

### Social determinants of health and health inequalities

In this section, we present the major SDH of Hispanic health including demography, socioeconomic status, environment, occupation, and mobility. We additionally discuss the significant contributions of culture, language, poverty, and gender to Hispanic health inequalities.

#### Demography

**Fig. 3:**
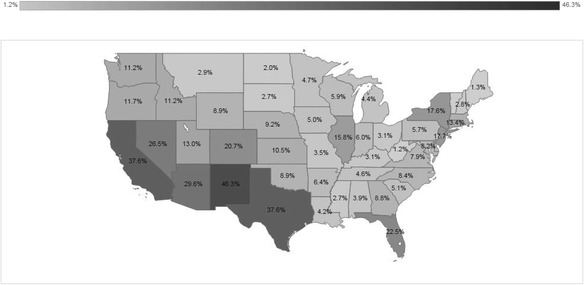
Percentage of Hispanic population in 2010**.** Source: [178]

Hispanics in the USA include people of Mexican (67.9%), Puerto Rican (10.1%), Salvadoran (4.0%), Cuban (3.9%), Dominican (3.4%), Guatemalan (2.6%), and other Hispanic origins (8.0%) [1]. In 2010, five US states had the largest Hispanic populations: California (14 million), Texas (9.5 million), Florida (4.2 million), New York (3.4 million), and Illinois (1.2 million). The following states had the highest proportion of Hispanic residents: New Mexico (46.3%), California (37.6%), Texas (37.6%), Arizona (29.6%), and Nevada (26.5%) (Fig. 3).

The median age of the US Hispanic population is 27 years, as compared to the US population (37 years) and NHWs (42 years) [29, 30]. Life expectancy at birth is longer for Hispanics (79.1 years for males, 83.8 for females) than for NHWs (76.5 years for males, 81.2 for females) (23), and Hispanics have a lower lifespan variability than do NHWs [31]. The growth of the US Hispanic population has created a significant racial/ethnic generational gap in the USA; in 2010, 34.9% of Hispanics were under the age of 18 compared to 20.9% of NHWs [32]. The percentage of Hispanics aged 65 and older is expected to grow by 134% between 2012 and 2050 as compared to 58.4% for the NHW population [33, 34].

#### Environment

About 91% of US Hispanics reside in metropolitan areas [35], and 28.3% of them reside near major highways with high traffic volume [36]. Living near a major highway is associated with adverse outcomes including acute [37] and chronic [38, 39] respiratory illnesses, cardiovascular diseases [40, 41], obstetrical complications [42], and poor pregnancy outcomes [43]. Cancer risk pollutants emitted indoors tend to be higher in Hispanic households [44].

Despite significant improvements in water availability and quality in the USA, some Hispanic communities still face water quality associated health threats including elevated levels of arsenic [45] and nitrates [46].

In 2013, 21.5% of US Hispanics were estimated to live near Superfund^1^ sites [47]. Compared to NHWs, Hispanics are more likely to reside in areas with increased industrial pollution [48]. Residence near hazardous waste sites has been positively related to an increase in hospitalization from diabetes [49].

#### Occupation

The employment rate among Hispanics (66.4%) is similar to that of NHWs (64%). Hispanic workers perform a disproportionate amount of unskilled, high-risk jobs (59%) as compared to NHWs (38.1%) in construction, domestic maintenance and repair services, nondurable manufacturing, and personal and household services [50]. Hispanics face an increased risk of mortality from some occupational hazards. As compared to NHW, the relative risk (RR) of a heat-related death among Hispanic agricultural workers was 3.4 (95% CI 2.0, 5.8) and among construction workers 1.7 (95% CI 1.1, 2.6); the risk of death from occupational carbon monoxide exposure was 1.4 (*p* < 0.05) [51, 52].

#### Mobility

**Fig. 4:**
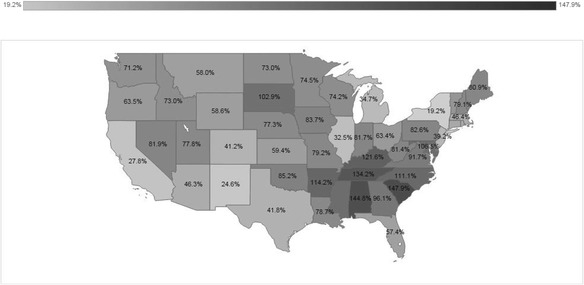
Percentage of Hispanic population growth 2000–2010. Source: [178, 179]

In recent years, the US Hispanic population has disseminated towards Central and Eastern States, with nine of them experiencing increments over 100% (SC, AL, TN, KY, AR, NC, MD, MS, and SD) in search of work and better living conditions (Fig. 4).

#### Culture and language

Hispanics in the USA have a particular social and cultural identity that characterizes them as an ethnic group. These, together with language, are the main manifestations of their culture. First-generation Hispanics (meaning they themselves were foreign-born) differ from second-generation Hispanics (US-born sons or daughters of at least one foreign-born parent) in language use, acculturation, diet, and other characteristics which exert differential health effects. For example, second- and third-generation Hispanics have an increased frequency of overweight and obesity when compared to first-generation Hispanics [53]. *Familism* is also an important cultural value among Hispanics [54] and is a source of emotional and financial support that may exert health risk-mitigating effects [17, 55].

In 2014, 68.4% of Hispanics reported they speak English at home or that they speak English “very well” as compared to 59.0% in 1980 [56]. Though English proficiency and educational level among Hispanics have increased, educational attainment is still low mainly among foreign-born Hispanics [57]. Between 2000 and 2013, the high school dropout rate decreased from 32 to 14%; still, it was nearly three times higher than that of NHWs (5%). Two- and four-year college enrollment increased 201% for Hispanics (versus 14% for NHWs), but in 2013 only 15% of Hispanics had at least a bachelor’s degree as compared to 40% of NHWs (34). Hispanics are underrepresented in Science, Technology, Engineering, and Mathematics (STEM) careers [58], and there is a growing shortage of Hispanic health care professionals [59].

#### Poverty and household income

In 2014, the median household income of Hispanics (USD $39,600) was 52% lower than that of NHWs (USD $60,300) [60]. Between 2000 and 2010, the poverty rate among Hispanics in the USA increased 5.1% as compared to that of NHWs (2.5%) [61]. By 2014, 23.6% of Hispanics were living below the poverty level, starkly differing from that of the national population (14.8%) and NHWs (10.1%). Additionally, the income-to-poverty ratio, a measure of depth of poverty, showed that 9.6% of Hispanics had income below one half of their poverty threshold (deep poverty) as compared to 4.6% of NHWs [60]. Poverty is high among the youngest and oldest Hispanics. In 2010, 6.1 million Hispanic children were living in poverty, the majority of which (4.1 million) were children of immigrant parents. Among adults 65 years and older, Hispanics have the highest poverty rate (20%) when compared to national estimates of poverty (10%) [62].

#### Poverty and health care

Health care services both influence health and are influenced by health needs. In the USA, health insurance is a key determinant of access to health care services. Hispanics have lower rates of health insurance enrollment than NHWs [63], a figure that is higher for recent immigrants [64]. In 2014, 26.5% of Hispanics were uninsured as compared to 10.4% of non-Hispanics under age 65. The gap was higher for persons aged 65 and over: 4.4% among Hispanics, compared with 0.5% among NHWs. When poverty levels were considered, gaps were higher. Among the Hispanic poor under age 65, 37.1% lacked insurance as compared to 19.7% of poor NHWs and 19.5% of poor African-Americans. Among persons aged 65 and over, 7.1% lacked health insurance as compared to 0.5% of near poor NHWs and 2.2% of poor African-Americans [65].

#### Gender

Health inequalities are heightened among US women, mostly related to social determinants such as unfair paid labor, schooling, and violence. Longitudinal analyses of changes in racial and gender inequality during the last four decades (1970 to 2010) have found important differences in median income by race/ethnicity among those aged 25 to 64 years. For example, in 1970 NHW women’s earned income was 20% higher than that for Hispanic women; it was 50% higher in 2009. Lower educational attainment among Hispanic women has worsened over time, from 11% in 1969 to 22% in 2009 [66]. Lower educational attainment has been shown to protect against morbidity, mortality, and depression, although recent research has shown that, among US-born Mexican-American women, higher educational attainment was associated with diabetes [67].

Hispanic women tend to be more vulnerable to abuse and mistreatment; Hispanic women may suffer lower self-esteem and higher intimate partner violence (IPV), with differential rates by country of origin [64]. Those who recently immigrated to the US—who work in bars or cantinas—were found to be at higher risks of experiencing intimate partner violence, including increased sexual risk behaviors from their primary or non-primary sexual partners [68].

#### Migrant seasonal farm workers (MSFW)

The US Hispanic population includes a large number of migrant and temporary workers who are foreign-born. Self-selection of migrant workers may explain some of the apparent health advantages in the US Hispanic population. This is also known as the *healthy migrant effect* [69].

MSFWs are one of the most marginalized populations in the USA. A high proportion of them (68%) were born in Mexico [70], 78% are males, 59% are married, and their average age is 36 years.

Given the nature of agricultural work, MSFWs face particular occupational health hazards such as pesticide exposure [71, 72], heat exposure [73], musculoskeletal injuries [74], respiratory illnesses [75], skin disorders [76], eye injuries [77], food insecurity [78], and depression [79]. Assessment of these and other health risks and outcomes is hampered by MSFWs’ highly mobile lifestyle, limited English proficiency, varying levels of citizenship status, and cultural barriers.

#### Undocumented immigrant workers

Unauthorized immigration is an important demographic phenomenon in the USA; undocumented immigrant workers play an important role in the US economy. Although declining, by 2012 the number of unauthorized immigrants was estimated at 11.2 million, the majority of whom (53%) were Mexican-born [80]. Undocumented migrant workers are employed in substandard, high-risk jobs with risky occupational exposures and very limited or no health insurance [81].

### Risk factors

This section presents some of the main risk factors underlying the most important chronic diseases affecting Hispanics in the USA, namely obesity, tobacco smoking, and alcohol intake.

#### Obesity

The obesity epidemic underlies multiple health issues among Hispanics; it is a common denominator in the development of metabolic syndrome, non-alcoholic fatty liver disease (NAFLD), diabetes, and cardiovascular disease (CVD). In addition, obesity increases the risk for several forms of malignancies [53]. The Hispanic population in the USA is disproportionately affected by obesity, with 42.5% [82] of adults currently classified as obese. This reflects a significant increase in prevalence since 1999, when approximately 20% of Hispanic adults were classified as obese [83]. Additionally, Hispanics have the highest rates of obesity among American youth (ages 2–19 years) at 21.9%, compared with 14.7% in NHWs [82], and Hispanic children aged 2–5 years have a fivefold higher prevalence of obesity compared to their NHW counterparts [82, 84]. Previous data showed that Hispanic children born outside of the USA were less likely to be obese than those born in the USA to immigrant parents [85]. More recent data demonstrate that foreign-born children of Hispanic immigrants are more likely to be overweight than children of more settled Hispanic immigrants and children of US natives [86].

The prevalence of obesity is heterogeneous among Hispanic subgroups, though across all subgroups females are more likely to be obese than males. Obesity rates vary from 26.8% of South American males to 51.4% of Puerto Rican females [87]. Puerto Rican Hispanics have a higher-risk profile for diabetes, cancer, and CVD [87]. Significant differences in the prevalence of obesity have also been noted between US-born Hispanics (47.1%) and foreign-born Hispanics (36.3%) [7]. Higher degrees of acculturation correspond with greater body weight in all migrant groups to the USA [88], though this effect is particularly pronounced in Mexican-born individuals [89, 90].

Obesity increases the risk for multiple associated health conditions. Obesity indirectly increases the risk of CVD and stroke by increasing the risk of hypertension [88] and diabetes [91]. The prevalence of diabetes and hypertension has been demonstrated to rise steadily in Hispanics of all ages with an increasingly elevated body mass index (BMI) [92]. Obesity also contributes to metabolic syndrome, which is characterized by insulin resistance. In turn, insulin resistance is a major risk factor for the development of diabetes as well as NAFLD [93], a condition that disproportionately affects Hispanics and can increase the risk of liver malignancies. Finally, the metabolic syndrome directly promotes the development of atherosclerotic CVD [94].

The high incidence of obesity in US Hispanics is a multifactorial problem. Food and beverage marketing for Hispanics in the USA promotes the consumption of low-nutrient, calorie-dense foods and beverages, especially among children [20]. Low-income Hispanic mothers have been found to engage in highly permissive, indulgent feeding patterns that relate directly to child obesity [95]. Food insecurity (when members of a household experience reduced quality, variety, or desirability of food products) has been significantly associated with obesity in low-income Mexican-American women living in California [96]. Other risk factors such as glucose intolerance and gestational diabetes affect Hispanic women and their descendants, as they will be more likely to develop diabetes themselves [97]. Moreover, health care inequalities contribute to obesity as well. For example, only 23% of Hispanics reported that their physician had discussed diet and exercise with them in the previous year; this percentage was lower (17%) among foreign-born Hispanics [91]. Behavioral factors have a much greater impact on premature death than does health care, making this lack of preventative counseling significant [98].

#### Tobacco

The tobacco industry targets Hispanics by utilizing custom advertising and by financially contributing to Hispanic community activities [99, 100]. In 2013, 20.9% of Hispanic adults in the USA had used tobacco products within the last month, as compared to 28.5% of the non-Hispanic population. The incidence of tobacco use is highest in Puerto Ricans, with 34.7% of males and 31.7% of females reporting tobacco use. In contrast, the incidence of smoking is lowest in Dominican males (11.1%) and Central American females (8.7%) [87].

In the USA, second-generation Hispanics have a disproportionately high rate of tobacco use, which increases their risk for CVD, diabetes, and cancer [101].

Many prevalent cancers in Hispanics (lung, breast, colorectal, and liver) share preventable risk factors, including, tobacco consumption, sedentary lifestyle, alcohol abuse, obesity, and an unhealthy diet. Additionally, other cancers (gastric, liver, and cervical) are associated with previous preventable or treatable infections, such as *Helicobacter pylori*, hepatitis B/C virus (HBV/HCV), and human papillomavirus. In the future, cancer mortality rates may decrease by avoiding risk factors that are the outcomes of acculturation, culturally insensitive public health approaches, and limited health care access [102].

#### Alcohol

Consumption of alcohol constitutes a risk factor for cancer, diabetes, CVD, and metabolic syndrome [103]. In the USA, Hispanics are less likely to binge-drink (defined as having a blood alcohol concentration greater or equal to 0.08 g/dL after 2 h of intake) when compared with NHWs [103]. However, alcohol consumption among Hispanics who already drink is higher than among NHWs. In 2010, the rate of alcohol dependence by country of origin was as follows: Puerto Rico 5.5%, Mexico 4.7%, South/Central America 3.1%, and Cuba 2.4% [90]. Binge drinking contributes to the development of fatty liver disease [104, 105].

### Morbidity and mortality

Social, environmental, and biological forces have modified the epidemiologic profile of Hispanics in the USA, with cancer being the leading cause of mortality, followed by cardiovascular diseases, liver disease, and unintentional injuries. CVD and diabetes share a host of common risk factors. Most specifically, these take the form of the metabolic syndrome, which is diagnosed when an individual meets any three of the following five criteria: elevated waist circumference (central obesity), elevated triglycerides, reduced high-density lipoprotein-C, elevated blood pressure, or elevated fasting glucose [94].

#### Cardiovascular disease

CVD is the second leading cause of death for Hispanics residing in the USA [67]. Significant risk factors for CVD include hyperlipidemia, tobacco use, diabetes, obesity, and hypertension [87]. Despite having an increased prevalence of several of the risk factors for CVD, Hispanics have a 25% lower death rate from cardiac disease than NHWs [7] and a 20% lower age-adjusted prevalence of congestive heart disease than NHWs [106]. This seemingly paradoxical finding may be explained by the relatively low median age of Hispanics residing in the USA, or it may represent an extension of the Hispanic Mortality Paradox, as supported by recent publications [7, 106, 107].

#### Diabetes

**Fig. 5:**
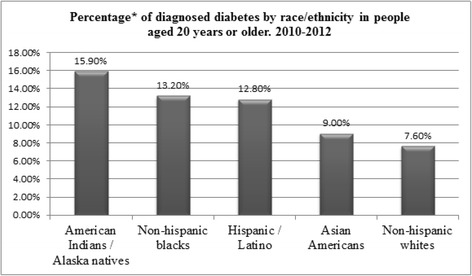
Percentage of diagnosed diabetes by ethnicity in people aged 20 years or older for the period 2010–2012. *Age-adjusted based on the 2000 US standard population. Source: [97]

**Fig. 6:**
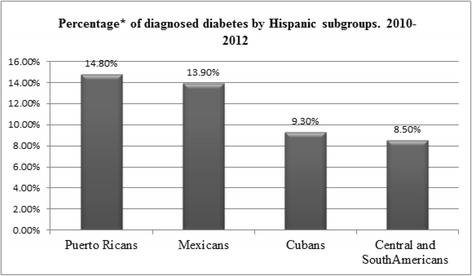
Percentage of diagnosed diabetes by Hispanic subgroups 2010–2012. *Age-adjusted based on the 2000 US standard population. Source: [97]

In 2012, it was estimated that 29 million Americans had diabetes [97]. The incidence of diabetes increased until 2010 and then decelerated between 2011 and 2014. As shown in Fig. 5, the Hispanic population is disproportionately affected by diabetes [97]. The incidence of diabetes in Hispanics has been increasing when compared to NHWs [108]. The prevalence of diabetes varies among Hispanic subgroups: in 2012, the age-adjusted rate of diagnosed diabetes was 14.8% for Puerto Ricans, 13.9% for Mexicans, 9.3% for Cubans, and 8.5% for Central and South Americans (Fig. 6) [97].

Hispanics with diabetes in the USA are affected by related comorbidities such as CVD, diabetic retinopathy, chronic renal disease, and diabetic neuropathy. These diseases generate additional medical expenses that especially affect uninsured Hispanics [97]. In the USA, the total diabetes expenditure for the year 2012 was 245 billion US dollars: 176 billion expended in direct medical costs (comorbidities, medications, medical supplies, hospitalization) and 69 billion in indirect costs (loss of employment, permanent disability, low healthy life expectancy) [97]. The medical expenses of Americans with diabetes were 2.3 times higher than the medical expenses of their healthy counterparts [97].

In the USA, diabetes mortality disproportionately affects Hispanics. In 2010, diabetes was one of the top ten causes of mortality in the country with 69,071 deaths [97]. The death rates from diabetes were significantly different for Hispanics (26.3 per 100,000) when compared to NHWs (18.6 per 100,000) in 2013. Among Hispanic males, diabetes death rates were higher (30.4 per 100,000) when compared to NHW males (23.1 per 100,000). For Hispanic women, diabetes death rates were also higher (23.0 per 100,000) when compared to NHW women (14.9 per 100,000) [108, 109].

#### Cancer

While Hispanics have a 30% lower mortality and 20% lower morbidity due to cancer than NHWs, cancer is now the leading cause of death among Hispanics. In 2015, 125,900 incident cases of cancer were estimated to have occurred among Hispanics, with a case-fatality rate of 30% (37,900 deaths) [110, 111]. Consistent with decreasing trends in cancer mortality among NHWs, cancer mortality has also decreased among Hispanic men (1.5% per year) and women (1.0%).

Cancer of the prostate is the most common cancer in Hispanic men (22%), followed by colorectal (11%) and lung (9%) cancer. Among women, breast cancer is the most frequent (29%), followed by thyroid (9%), colorectal (8%), and uterine (8%) cancer. Among men, the main cause of cancer death is lung (17%) cancer, followed by liver (12%) and colorectal (11%) cancer [112]. Among women, the most frequent cause of cancer death is breast (16%) cancer, followed by lung (13%) and colorectal (9%) cancer. Breast cancer death rates are about 30% lower in Hispanic women than NHW women. The incidence of gastric cancer is increasing in young Hispanic men [113, 114].

Cancer morbidity and mortality rates differ by country of origin and ethnicity. For example, Puerto Ricans and Cubans have similar incidence rates as NHWs but lower rates than Mexicans. Death rates among Puerto Ricans are 12% lower than for NHW men but 20% higher than those of Mexican men. Compared to NHWs, Hispanics are more likely to be diagnosed with infection-related cancers such as gastric, hepatic, and cervical but are less likely to be diagnosed with prostate, breast, lung, and colorectal cancer. US-born male Hispanics are twice as likely to develop hepatocellular carcinoma than foreign-born Hispanics [115]. The reasons for these morbidity and mortality differences have been attributed to first-generation status, healthy immigrant effects, country of origin, younger age structure, late-stage diagnoses, and lower survival rates likely due to lower access to preventive and diagnostic health services [112, 116].

#### Liver disease

Liver disease is the 11th most common cause of death in Americans, but the sixth most common cause of death in Hispanic individuals residing in the USA. For both Hispanics and NHWs, deaths attributed to chronic liver disease are equally divided between alcohol and other causes [7]. Hispanic individuals in the USA have a 48% higher death rate from liver disease and cirrhosis than NHWs. Common causes of liver disease affecting Hispanics in the USA include non-alcoholic fatty liver disease (NAFLD), alcoholic liver disease (ALD), and chronic infections with viral hepatitis.

NAFLD encompasses a variety of liver conditions that are histologically similar to alcoholic liver disease and can culminate in cirrhosis and liver failure [93]. Obesity and insulin resistance, two components of the metabolic syndrome, are risk factors for NAFLD and have been found to be correlated with hepatic fat accumulation in Hispanics [117]. NAFLD is associated with CVD in all affected populations, and cardiovascular complications are the most common cause of death in persons with NAFLD [118, 119]. The worldwide prevalence of NAFLD is estimated at 20%. The prevalence of NAFLD in Hispanics living in the USA is at least 29% [120, 121], where the risk is heterogeneous. For instance, Hispanics of Mexican origin maintain a significantly higher risk of NAFLD (33%) than do individuals of Dominican (16%) or Puerto Rican (18%) origin [12].

The risk for Mexican-Americans remains high even after controlling for age, sex, BMI, waist circumference, hypertension, and insulin resistance. The reasons for the Hispanic preponderance of NAFLD have yet to be fully elucidated; polymorphisms in the patatin-like phospholipase domain-containing 3 gene, which is exceptionally common in Hispanic populations, may help to explain the susceptibility to NAFLD [93, 122, 123].

There is a paucity of literature regarding ALD among US Hispanics. Overall, Hispanics have lower rates of alcohol consumption than do NHWs. Mexican and Puerto Rican subgroups have the highest documented alcohol consumption [124]. There are disparities in care which affect Hispanics, including decreased access to professional substance abuse programs [121]. Hispanic patients with ALD often present with more severe disease at earlier ages than do NHW and Black patients [125].

The contribution of viral hepatitis to liver disease in the Hispanic population is similar to that of the general NHW population. The Hispanic Community Health Study/Study of Latinos (HCHS/SOL) reported that the overall incidence of active HBV infection was 0.29% [126]. However, these results were heterogeneous, with the highest incidence of active infection noted in those born in the Dominican Republic (0.95%) and the USA (0.57%). The HCHS/SOL cohort also demonstrated heterogeneity in HCV infection among Hispanics living in the USA [126].

The HCV prevalence among Hispanics 18–74 years of age residing in the USA was reported as 1.5% in the National Health and Nutrition Examination Survey 2007–2010 [4, 127] and 2.0% in the HCHS/SOL population [128]. The HCV seropositivity rates varied from 0.4% among males of South American descent to 11.6% of males of Puerto Rican descent. On average, females had lower rates of HCV than males in all Hispanic subgroups. The rates of HCV also varied by age and current city of residence [126]. All of the previously mentioned causes of liver disease increase the risk of liver cancer. US Hispanics are also at higher risk of developing [3] and dying from cancer of the liver and bile ducts [7, 129].

#### Unintentional injuries

Mortality from unintentional injuries in 2010 ranked third for the Hispanic population, accounting for 7.3% of deaths after malignant neoplasms (21.5%) and heart disease (20.8%), while deaths from unintentional injuries accounted for 4.8 and 4.2% among NHWs and non-Hispanic blacks, respectively [9].

The three leading causes of unintentional injuries were motor vehicle collisions, accidental poisoning, and falls. When combined, these three represented 49.2 and 50.2% of all deaths from intentional and unintentional causes in 2000 and 2009, respectively [130]. Furthermore, Hispanics and Blacks had lower motor vehicle traffic crash adjusted mortality rates than did Whites. These are in line with previous findings examining ethnic differences showing declines in unintentional injury deaths among Hispanics as compared to NHWs for the periods 1992–2002 [131] and 1999–2005 [132]. Lastly, increased pedestrian-related deaths in the Hispanic elderly population contribute to mortality from unintentional injuries in this vulnerable population [133].

### The Latina Birth Outcomes and the Hispanic Mortality Paradoxes

Despite their socioeconomic disadvantages and burden of disease, all-cause mortality among Hispanics is 24% lower than for NHWs and mortality is also lower for nine out of 15 leading causes of death [7]. Hispanics in the USA have a longer life expectancy at birth and experience some better health outcomes than similar socioeconomic groups, a phenomenon described roughly 30 years ago as the “Hispanic Mortality Paradox.” Seeming to persist to this date, the Hispanic Mortality Paradox is mostly attributed to a “healthy migrant effect” [134–137] involving cultural mores and health behaviors of first-generation Hispanics in the USA. Additionally, Hispanic women in the USA have birth outcomes similar to those of women with a higher socioeconomic status and access to health services in the USA, a phenomenon known as the “Latina Birth Outcomes Paradox” [138, 139], apparently due to acculturation-related behaviors such as diet, smoking, and social support [18], although this paradox remains controversial [140, 141].

#### Infant mortality and the Latina Birth Outcomes Paradox

The infant mortality rate is an accurate reflection of a nation’s health that proves that social determinants of health are shaped by the economics, social policies, and politics surrounding the circumstances in which people are born, grow up, live, work, and age [142].

The US Centers for Disease Control and Prevention (CDC) estimates the infant mortality rate in the USA at 5.96 per 1000 live births, which is only a 10% reduction since the year 2000. Specifically, for Hispanics the infant mortality rate is 5.1 per 1000 live births [143]. Hispanics in the USA have the highest birth rate among racial/ethnic groups [144]. Despite being a vulnerable population due to their socioeconomic status and inadequate health care, babies born to Hispanic women, particularly to foreign-born mothers, experience lower rates of low birth weight and mortality compared to national averages, a phenomenon known as the Latina Birth Outcomes Paradox [145–147]. Various explanations have been proposed for this paradox. One is that perceived cultural and protective factors may be a result of social support from extended family members, community health workers, and lay midwives [148]. Some protective factors that have been identified “include a strong cultural support for maternity, healthy traditional dietary practices, and the norm of selfless devotion to the maternal role” [149]. However, given the health coverage disadvantage in this population, the potential for their undocumented/inadequate legal status presents challenges to the foreign-born Hispanic pregnant population. While the importance of adequate prenatal care is recognized, as seen in the Latina Paradox, it shows that there is more to prenatal care in this population.

The CDC states that “the risk of…infant mortality and pregnancy-related complications can be reduced by increasing access to quality…care [because pregnancy provides] an opportunity to identify existing health risks in women and to prevent future health problems for women and their children” [150].

An unanswered question is whether the ACA and enhanced Medicaid perinatal care programs are having an effect on birth outcomes [151]. Initial data are promising that the ACA does indeed have the capacity to improve perinatal outcomes even further once fully implemented, though these data are not yet available [151]. Prematurity, the number one reason for infant mortality, declined in the states that have implemented Medicaid expansion [152].

Nonetheless, the main reasons for Hispanic women not being able to access care are (1) not being “poor enough” to qualify for Medicaid without any structured perinatal care on which to rely on; (2) unable to afford coverage offered by the ACA; and (3) born outside the USA and may not qualify for Medicaid coverage depending on their legal status and the state they live in, as there are differences in eligibility and perinatal coverage among states [153]. It thus begs the question as to how Hispanic women within the gap will be able to afford and obtain quality perinatal care.

#### The Hispanic Mortality Paradox

The Hispanic Mortality Paradox refers to lower mortality rates and better health outcomes among foreign-born, newly arrived, and thus less acculturated Hispanics as compared to native-born Hispanics or to NHWs. Reasons for this paradox may be that migrants and first-generation Hispanics may represent a self-selected healthier population. Also, undocumented or un-acculturated Hispanics may leave the country if unhealthy or their negative health outcomes may be underreported due to lack of access to health services. Lower rates of smoking seem to be at the heart of the Hispanic Mortality Paradox; however, the obesity and diabetes epidemics, together with higher levels of inflammatory biomarkers and increasing social and environmental stressors, may offset the health advantages of Hispanics in the future [106, 107, 135, 136].

### Health services

The social response to health needs is implemented through health policy and programs, generically called “health services.” Typical health service indicators include access, coverage, utilization, costs and expenditures, and quality and performance. In the case of Hispanics, health literacy and cultural competence play important roles.

The implementation of the ACA has increased access to health care for Hispanics; a recent survey showed that 87% of US-born Hispanics have health insurance, compared to 78% of those born outside the USA [154]. The ACA has expanded coverage by 5.3% since it was established, granting access to Medicaid and state and federal health insurance. The uninsured rate has decreased by 11.9% among Hispanics compared to a 6.1% decrease among NHWs [155]. However, barriers to access persist: about half of Hispanics who are uninsured have household incomes under 133% of the poverty line (about USD $15,500 a year), which makes them eligible for Medicaid [10, 156]. As of December 10, 2015, 25 states had expanded Medicaid while 20 had not and another six are using alternative expansion waivers.^2^ By the end of 2014, about a quarter of Hispanics remained uninsured in states that expanded Medicaid eligibility as compared to about a half of Hispanics in states that have not, and still higher percentages remain uninsured in states like Texas and Florida, which have the second and third largest Hispanic populations.

An analysis of four national health surveys reported higher rates (41.5%) of uninsured Hispanics ages 18–64 years (45.3% men, 37.4% women) compared with 15.1% of NHWs of the same age. Moreover, foreign-born Hispanics 18–64 years were over twice as likely to be uninsured than US-born individuals (54.7 vs. 25.9%, respectively). About 15.5% of Hispanics reported delayed or lack of medical care because of cost concerns compared to just 13.6% of NHWs and 12.5% did not obtain needed prescription drugs because of cost compared to just 9.5% of NHWs [7].

Hispanics use fewer health screenings, have less follow-up care, and face more economic and cultural barriers to health care. Hispanics are 28% less likely to be screened for colorectal cancer than are NHWs. Hispanic women have less access to breast cancer and cervical cancer screening [157, 158].

Hispanics are more likely than NHWs to receive mental health care through emergency departments rather than self-referral or outpatient services [159]. They are also more likely than other ethnic groups to discontinue diabetes medications after losing health care coverage [160].

The number of Hispanic health care professionals does not mirror the total percentage of Hispanics in the USA (17.4%). Hispanic professionals have been historically underrepresented in the health professions in the USA: pharmacists make up 3.4%, physicians 5%, physician assistants 3.7%, licensed registered nurses 1.7%, and dentists 3.3% of health professionals [161]. In 2015–2016, medical enrollment and graduation remains at about 5%.

Hispanics comprise about 14% of the total US workforce in the USA. About 50% of the 22 million Hispanic workers in the USA are immigrants. Immigrant workers are often employed in high-risk jobs where they bear a high burden of occupational injuries, often holding temporary jobs with no health benefits. They are also often geographically mobile, thus unable to have a usual care provider and obtain continuity of care [162]. Undocumented Hispanics make up about 5% of the US workforce but they are ineligible for health insurance and thus more likely to advance to severe illness and use emergency care services. A standardized approach to providing emergency care is through the Emergency Medical Treatment and Labor Act, a long-standing act of Congress that “imposes specific obligations on Medicare-participating hospitals that offer emergency services to provide a medical screening examination when a request is made for examination or treatment for an emergency medical condition, including active labor, regardless of an individual’s ability to pay” [163].

### Recommendations

This scoping review provides an updated account of the social determinants of health, health inequalities, and risk factors shaping Hispanic morbidity and mortality trends in the one hand and the organized social response by health services in the other. Our review considers recent information on Hispanic subpopulation types: Hispanics born in the USA, foreign-born, undocumented, and migrant and seasonal farm workers (MSFW). The increasing heterogeneity of the Hispanic population in the USA by country of origin is also taken into account. Additionally, we provide brief updates on the Hispanic Mortality Paradox and the Latina Birth Outcomes Paradox.

A multilevel, multifaceted approach, from social policy to health services, is needed to improve the health of Hispanics in the USA. We identified three priority policy and programmatic areas to be pursued.

#### Adopt a Health in All Policies approach

Social, environmental, and biological forces have modified the epidemiologic profile of Hispanics in the USA [164]. Health in All Policies is an initiative to frame collaborative approaches among all social sectors towards embedding health and equity into government decision-making processes [165]. In the USA, the Healthy People initiative provides science-based, 10-year national health promotion and disease prevention goals. Healthy People 2020 [150] goals for Hispanic health programs should target improving access to healthier food choices, preventing environmental exposures, offering safe environments for exercise and recreation, and increasing access to primary and preventative health care access.

Initiatives like those proposed by the Social Determinants of Health Work Group at the CDC offer a roadmap to address SDHs and health inequalities through five key domains: (1) economic stability, (2) education, (3) health and health care, (4) neighborhood and built environment, and (5) social community context. They identify national, state, and local resources and point out national experiences of interventions to improve social determinants of health [166].

Intersectoral actions are key to address the diversity of social determinants of health and also involve partnering with communities to engage them and increase the pertinence of interventions. The interconnected nature of determinants of health, health inequalities, and risk factors herein presented require equally comprehensive initiatives that would not only target and help Hispanics but other ethnic and vulnerable populations sharing similar contexts, through multidisciplinary, multisectoral programs aiming at generating sustainable local capacities [166].

The integrated approach presented in our conceptual framework reflects the opportunity for the different social sectors to share information and collaborate with direct actions targeting the different social determinants of health within their area of responsibility. For example, health authorities can collaborate with housing, urban development counterparts to generate comprehensive programs focusing on improving local housing and the built environment, as well as indoor environmental conditions. Table 1 presents some of the most prominent organizations and initiatives working on improving Hispanic health in the USA.

#### Increase health care access

Unequal health insurance markets have created a variegated array of health care access for Hispanics in the USA. It is unclear how the final implementation and shape of Medicaid eligibility provided by the ACA will benefit Hispanics. Major obstacles to health care access for Hispanics should be addressed, particularly those originating from substandard employment with limited health benefits, limited number of Hispanic health care providers, cultural sensitivity, geographic mobility, and undocumented status. These barriers result in Hispanics using fewer therapeutic and preventive health services even with increased access to, and utilization of, health services through the ACA [167, 168].

The underrepresentation of Hispanic health care workers in health professions programs must be addressed through pipeline education programs, affirmative action admissions, diversity requirements for school accreditation, tuition loan repayment programs and federal, and scholarship funding to support diversity enrichment programs. Similar Hispanic representation must be accomplished at all levels of decision-making and services, following the Health in All Policies framework outlined above, to respond to the changing demographic and health profiles of Hispanics in the USA [169].

Enhancing cultural sensitivity and health literacy is needed to increase Hispanics’ access to and utilization of health services, particularly for controlling chronic diseases, fostering healthy lifestyles, obesity prevention, workplace safety, and utilization of preventive and screening services [170]. Spanish-speaking health providers have been shown to improve control of chronic diseases and improve patients’ adherence to health recommendations and patient satisfaction [171]. In the USA, health care professionals are required by law to offer language translation and interpretation services to individuals with Limited Language Proficiency (LEP)—defined as “LEP language group that constitutes 5% or 1000 persons, whichever is less, of the population served.” Title IV of the Civil Rights Act of 1964 considers failure to provide these services discriminatory and results in losing eligibility status for federal funding of health services. This was expanded in 1997 through the Critical Access Hospital Program which requires that documents such as eligibility criteria for services, informed consent documents, discharge instructions, complaint forms, and other documents are provided in the language of LEP individuals; enforcement of these regulations is the responsibility of the Department of Health and Human Services Office for Civil Rights [172].

Migration forces to the USA are diverse; multilateral country collaborations between migrant sender and recipient communities are needed to develop health care programs for Hispanics. Immigrants’ access to health services varies among states according to legal status, country of origin, and cultural and linguistic issues. For example, Puerto Ricans have citizenship rights in the USA and refugees and asylees in the USA are granted Medicaid coverage in the USA [173]. Undocumented immigrants are banned from purchasing health care services under the ACA. In June 2015, California passed a bill to allow undocumented immigrants to purchase ACA insurance [174]. Proponents of access to undocumented workers believe that allowing access to health care would reward this workforce for their contributions to society and advance social justice for this vulnerable population [19, 81]. International epidemiologic intelligence information must be shared across borders among migrant sender and recipient communities of migrant workers to prevent and respond to health risks [175].

#### Generate and disseminate knowledge

Efforts should continue to disseminate the results of health disparities research and promotion and risk prevention strategies among Hispanics. Research must capture and interpret the sociocultural factors to explain Hispanic health inequalities by improving the terminology to identify Hispanics [176] and increasing participation of Hispanics in health research. To this end, new research paradigms must use multilevel models and implementation science to incorporate the continuum of social determinants of health, health inequalities, and risk factors that modulate the epidemiologic profile of Hispanics in the USA. Research constructs must adapt to the changing dynamics of Hispanic demographics and social conditions, in addition to the effects of policy changes introduced by the ACA for eligible and ineligible Hispanics. Translating research findings into practice will require funding multidisciplinary collaborations between Hispanic community stakeholders, government, and non-governmental organizations [177].

## Conclusions

The complexity of factors impinging on Hispanic health requires addressing the social determinants of health related to the quality of the social and physical environment where Hispanics live and work, including neighborhoods, housing, transportation, and environmental and employment conditions.

The changing profile of Hispanic morbidity and mortality offers new opportunities to further address the main morbidity and mortality causes and further the health outcomes underlying the Hispanic and Latina Birth Outcomes Paradoxes by curbing the obesity epidemic, expanding antenatal and perinatal care, preventing and ceasing smoking, and decreasing workplace hazards.

Limited cultural sensitivity, health illiteracy, and a shortage of Hispanic health care providers remain as the main barriers to access to health services for Hispanics. Even for those with access to health care services, underutilization of preventive care is still a challenge. Migrant and undocumented workers are disproportionately exposed to health risks in the workplace, with limited access to health services.

Multiple gaps are evident regarding knowledge needed to improve Hispanic health. The weight of the evidence on Hispanic health is mostly from cross-sectional studies that offer nationwide averages, obscuring focalized health disparities and inequalities. The health of Hispanics in the USA differs by demographic, ethnic, and cultural subgroups. Understanding and addressing Hispanic health issues in a comprehensive way requires a targeted approach to country of origin and idiosyncrasy.

The framework and scoping methodology guiding this review allow a comprehensive approach to assessing and monitoring Hispanic health in the USA and may be replicated at the state and local levels to evaluate the impact of social and health policies.

## Additional file

Additional file 1: Supplemental Material. Review papers on Hispanic Health cited in PubMed from 2006 through September 2016. (DOCX 36 kb)

## Footnotes

1 “Superfund sites” are the most polluted hazardous waste sites managed by the US Environmental Protection Agency as cleanup areas, with potential threats to human health and the environment (https://www.epa.gov/superfund).

2 Federal requirement waivers to implement demonstration projects to pilot-test Medicaid eligibility, managed care, cost sharing, benefit packages, and other types of healthcare.
