# Supplementary material for: Hispanic health in the USA: a scoping review of the literature
Source: Public Health Rev. 2016 Dec 7;37:31. doi: 10.1186/s40985-016-0043-2 (PMC5809877; doi:10.1186/s40985-016-0043-2)
Supplement: Supplementary file 1 — Supplemental Material. Review papers on Hispanic Health cited in PubMed from 2006 through September 2016. (DOCX 36 kb) [file 40985_2016_43_MOESM1_ESM.docx]

SUPPLEMENTAL MATERIAL

Review papers on Hispanic Health cited in PubMed from 2006 through September 2016.

| Year (#) | Keywords |  |
| --- | --- | --- |
| 2006 (1) | Acculturation, cultural characteristics, gender identity, adolescent behavior, Health Knowledge, Attitudes, Practice | *Afable-Munsuz, A., & Brindis, C. D. (2006). Acculturation and the sexual and reproductive health of Latino youth in the United States: a literature review. Perspect Sex Reprod Health, 38(4), 208-219. |
| 2007 (9) |  |  |
|  | Environmental health; asthma, emigration and immigration, risk factors. | *Carter-Pokras, O., Zambrana, R. E., Poppell, C. F., Logie, L. A., & Guerrero-Preston, R. (2007). The environmental health of Latino children. J Pediatr Health Care, 21(5), 307-314. |
|  | Cardiovascular Diseases/ epidemiology/ etiology  Risk Factors | *Davidson, J. A., Kannel, W. B., Lopez-Candales, A., et al. (2007). Avoiding the looming Latino/Hispanic cardiovascular health crisis: a call to action. Ethn Dis, 17(3), 568-573 |
|  | Diabetes Mellitus, Type 2/ethnology/ prevention & control  Health Knowledge, Attitudes, Practice  Health Promotion/ methods | Latham, C. L., & Calvillo, E. (2007). A health protection model for Hispanic adults with Type 2 diabetes. J Clin Nurs, 16(7B), 186-196. |
|  | Interpersonal Relations  Leisure Activities  Melanosis/ diagnosis/ethnology/ therapy  Quality of Life | Pawaskar, M. D., Parikh, P., Markowski, T., McMichael, A. J., Feldman, S. R., & Balkrishnan, R. (2007). Melasma and its impact on health-related quality of life in Hispanic women. J Dermatolog Treat, 18(1), 5-9. |
|  | Community Health Workers/ education/utilization  Health Promotion/ manpower | *Rhodes, S. D., Foley, K. L., Zometa, C. S., & Bloom, F. R. (2007). Lay health advisor interventions among Hispanics/Latinos: a qualitative systematic review. Am J Prev Med, 33(5), 418-427. |
|  | Delivery of Health Care/trends  Diabetes Complications/epidemiology/ ethnology | Umpierrez, G. E., Gonzalez, A., Umpierrez, D., & Pimentel, D. (2007). Diabetes mellitus in the Hispanic/Latino population: an increasing health care challenge in the United States. Am J Med Sci, 334(4), 274-282. |
|  | Depressive Disorder/ prevention & control/ therapy  Disease Management  Ethnic Groups | Van Voorhees, B. W., Walters, A. E., Prochaska, M., & Quinn, M. T. (2007). Reducing health disparities in depressive disorders outcomes between non-Hispanic Whites and ethnic minorities: a call for pragmatic strategies over the life course. Med Care Res Rev, 64(5 Suppl), 157S-194S. |
|  | Maternal-Child Health Centers/ utilization  Preventive Medicine | Wasserman, M., Bender, D., & Lee, S. Y. (2007). Use of preventive maternal and child health services by Latina women: a review of published intervention studies. Med Care Res Rev, 64(1), 4-45. |
|  | Health Services Accessibility  Healthcare Disparities  Breast Neoplasms/ prevention & control | *Wells, K. J., & Roetzheim, R. G. (2007). Health disparities in receipt of screening mammography in Latinas: a critical review of recent literature. Cancer Control, 14(4), 369-379. |
| 2008 (5) |  |  |
|  | Healthcare Disparities  Hispanic Americans/ psychology  Mental Health Services | *Lopez, C., Bergren, M. D., & Painter, S. G. (2008). Latino disparities in child mental health services. J Child Adolesc Psychiatr Nurs, 21(3), 137-145. |
|  | Cultural Diversity  Emigration and Immigration  Health Behavior/ ethnology  Accessibility/organization & administration | Macnaughton, N. S. (2008). Health disparities and health-seeking behavior among Latino men: a review of the literature. J Transcult Nurs, 19(1), 83-91. |
|  | Healthcare Disparities/economics  Hispanic Americans/ psychology  Humans  Medically Uninsured  Mental Disorders/ethnology | Manoleas, P. (2008). Integrated primary care and behavioral health services for Latinos: a blueprint and research agenda. *Soc Work Health Care, 47*(4), 438-454. |
|  | Breast Feeding/ psychology  Diabetes Mellitus/diet therapy/prevention & control/psychology  Health Knowledge, Attitudes, Practice | *Perez-Escamilla, R., Hromi-Fiedler, A., Vega-Lopez, S., Bermudez-Millan, A., & Segura-Perez, S. (2008). Impact of peer nutrition education on dietary behaviors and health outcomes among Latinos: a systematic literature review. J Nutr Educ Behav, 40(4), 208-225. |
|  | Consumer Participation/ methods/psychology  Cultural Competency  Cultural Diversity  Emigrants and Immigrants | *Shelton, D. (2008). Establishing the public's trust through community-based participatory research: a case example to improve health care for a rural Hispanic community. Annu Rev Nurs Res, 26, 237-259. |
| 2009 (6) |  |  |
|  | Acculturation  Communication  Health Behavior  Health Promotion/ methods | *Elder, J. P., Ayala, G. X., Parra-Medina, D., & Talavera, G. A. (2009). Health communication in the Latino community: issues and approaches. Annu Rev Public Health, 30, 227-251. |
|  | Attitude to Health/ ethnology  Chronic Disease/ethnology  Cultural Characteristics  Health Behavior/ ethnology | *Gallo, L. C., Penedo, F. J., Espinosa de los Monteros, K., & Arguelles, W. (2009). Resiliency in the face of disadvantage: do Hispanic cultural characteristics protect health outcomes? J Pers, 77(6), 1707-1746. |
|  | Chronic Disease  Diabetic Nephropathies/ethnology  Disease Progression  Emigrants and Immigrants | *Lora, C. M., Daviglus, M. L., Kusek, J. W., Porter, A., Ricardo, A. C., Go, A. S., & Lash, J. P. (2009). Chronic kidney disease in United States Hispanics: a growing public health problem. *Ethn Dis, 19*(4), 466-472. |
|  | Breast Neoplasms/prevention & control  Community Health Services/ manpower  Community Health Workers/ utilization | *Martinez-Donate, A. P. (2009). Using lay health advisors to promote breast and cervical cancer screening among Latinas: a review. WMJ, 108(5), 259-262. |
|  | Acculturation  Emigrants and Immigrants/ statistics & numerical data  Health Status Disparities | Thomson, M. D., & Hoffman-Goetz, L. (2009). Defining and measuring acculturation: a systematic review of public health studies with Hispanic populations in the United States. Soc Sci Med, 69(7), 983-991. |
|  | Health Status Disparities | *Vega, W. A., Rodriguez, M. A., & Gruskin, E. (2009). Health disparities in the Latino population. Epidemiol Rev, 31, 99-112. |
| 2010 (5) |  |  |
|  | Counseling  Health Personnel  Health Promotion | *Ayala, G. X., Vaz, L., Earp, J. A., Elder, J. P., & Cherrington, A. (2010). Outcome effectiveness of the lay health advisor model among Latinos in the United States: an examination by role. *Health Educ Res, 25*(5), 815-840. |
|  | Diabetes Complications/ prevention & control  Diabetes Mellitus, Type 2  Healthcare Disparities  Medical Informatics Applications  Quality Improvement | *Baig, A. A., Wilkes, A. E., Davis, A. M., Peek, M. E., Huang, E. S., Bell, D. S., & Chin, M. H. (2010). The use of quality improvement and health information technology approaches to improve diabetes outcomes in African American and Hispanic patients. *Med Care Res Rev, 67*(5 Suppl), 163S-197S. |
|  | Cost of Illness  Health Promotion  Neoplasms/complications/ prevention & control/psychology/ virology | *Colon-Lopez, V., Ortiz, A. P., & Palefsky, J. (2010). Burden of human papillomavirus infection and related comorbidities in men: implications for research, disease prevention and health promotion among Hispanic men. *P R Health Sci J, 29*(3), 232-240. |
|  | Health Knowledge, Attitudes, Practice  Sleep Wake Disorders/ epidemiology | *Loredo, J. S., Soler, X., Bardwell, W., Ancoli-Israel, S., Dimsdale, J. E., & Palinkas, L. A. (2010). Sleep health in U.S. Hispanic population. *Sleep, 33*(7), 962-967. |
|  | Health Surveys  Kidney Failure, Chronic/ ethnology/psychology  Quality of Life | *Porter, A. C., Vijil, J. C., Jr., Unruh, M., Lora, C., & Lash, J. P. (2010). Health-related quality of life in Hispanics with chronic kidney disease. *Transl Res, 155*(4), 157-163. |
| 2011 (3) |  |  |
|  | Attitude to Health/ ethnology  Cultural Characteristics  HIV Infections/ ethnology/psychology  Health Education/ methods | *Instone, S., & Mueller, M. R. (2011). Religious influences on the reproductive health decisions of HIV-positive Latinas on the border. *J Relig Health, 50*(4), 942-949. |
|  | Acculturation  Disease Progression  Health Knowledge, Attitudes, Practice  Health Literacy | *Lora, C. M., Gordon, E. J., Sharp, L. K., Fischer, M. J., Gerber, B. S., & Lash, J. P. (2011). Progression of CKD in Hispanics: potential roles of health literacy, acculturation, and social support. *Am J Kidney Dis, 58*(2), 282-290. |
|  | Diabetes Mellitus, Type 2/epidemiology/ethnology/etiology  Diet/adverse effects  Health Status Disparities | *Perez-Escamilla, R. (2011). Acculturation, nutrition, and health disparities in Latinos. *Am J Clin Nutr, 93*(5), 1163S-1167S. |
| 2012 (4) |  |  |
|  | Contraception  Health Education/ methods  Health Knowledge, Attitudes, Practice  Health Promotion/ methods | *Cardoza, V. J., Documet, P. I., Fryer, C. S., Gold, M. A., & Butler, J., 3rd. (2012). Sexual health behavior interventions for U.S. Latino adolescents: a systematic review of the literature. *J Pediatr Adolesc Gynecol, 25*(2), 136-149. |
|  | Child Health Services/ organization & administration/standards  Child, Preschool  Cultural Competency  Delivery of Health Care/ organization & administration/standards | Kim-Godwin, Y., & McMurry, M. J. (2012). Perspectives of nurse practitioners on health care needs among Latino children and families in the rural Southeastern United States: a pilot study. *J Pediatr Health Care, 26*(6), 409-417. |
|  | Diabetes Complications/epidemiology/ethnology/prevention & control  Diabetes mellitus/epidemiology/ethnology  Health Status Disparities | *Lopez, L., & Grant, R. W. (2012). Closing the gap: eliminating health care disparities among Latinos with diabetes using health information technology tools and patient navigators. *J Diabetes Sci Technol, 6*(1), 169-176. |
|  | Health Services Accessibility  Healthcare Disparities/ ethnology/statistics & numerical data  Mental Disorders/ethnology/ therapy | Lopez, S. R., Barrio, C., Kopelowicz, A., & Vega, W. A. (2012). From documenting to eliminating disparities in mental health care for Latinos. *Am Psychol, 67*(7), 511-523. |
| 2013 (4) |  |  |
|  | Health Status  Lactose Intolerance/ ethnology  Morbidity/trends | Bailey, R. K., Fileti, C. P., Keith, J., Tropez-Sims, S., Price, W., & Allison-Ottey, S. D. (2013). Lactose intolerance and health disparities among African Americans and Hispanic Americans: an updated consensus statement. *J Natl Med Assoc, 105*(2), 112-127. |
|  | Cultural Characteristics  Health Literacy  Mass Screening/ utilization  Uterine Cervical Neoplasms | Flores, B. E., & Acton, G. J. (2013). Older Hispanic women, health literacy, and cervical cancer screening. *Clin Nurs Res, 22*(4), 402-415. |
|  | Choice Behavior  Health Care Reform  Health Services Accessibility/ statistics & numerical data  Healthcare Disparities/ statistics & numerical data | Guerrero, E. G., Marsh, J. C., Khachikian, T., Amaro, H., & Vega, W. A. (2013). Disparities in Latino substance use, service use, and treatment: implications for culturally and evidence-based interventions under health care reform. *Drug Alcohol Depend, 133*(3), 805-813. |
|  | Epidemics/statistics & numerical data  HIV Infections/epidemiology/ prevention & control  Health Promotion/ methods | *Sutton, M. Y., & Parks, C. P. (2013). HIV/AIDS prevention, faith, and spirituality among black/African American and Latino communities in the United States: strengthening scientific faith-based efforts to shift the course of the epidemic and reduce HIV-related health disparities. *J Relig Health, 52*(2), 514-530. |
| 2014 (11) |  |  |
|  | Attitude to Death/ ethnology  Cultural Competency  Palliative Care/ psychology  Professional-Family Relations | Adames, H. Y., Chavez-Duenas, N. Y., Fuentes, M. A., Salas, S. P., & Perez-Chavez, J. G. (2014). Integration of Latino/a cultural values into palliative health care: a culture centered model. *Palliat Support Care, 12*(2), 149-157. |
|  | Biomedical Research/ organization & administration  Needs Assessment  Patient Selection | *Aragones, A., Hayes, S. L., Chen, M. H., Gonzalez, J., & Gany, F. M. (2014). Characterization of the Hispanic or latino population in health research: a systematic review. *J Immigr Minor Health, 16*(3), 429-439. |
|  | Cardiovascular Diseases/ ethnology/etiology  Cohort Studies  Diabetes Mellitus/epidemiology/ethnology | Daviglus, M. L., Pirzada, A., & Talavera, G. A. (2014). Cardiovascular disease risk factors in the Hispanic/Latino population: lessons from the Hispanic Community Health Study/Study of Latinos (HCHS/SOL). *Prog Cardiovasc Dis, 57*(3), 230-236. |
|  | Cardiovascular Diseases/ethnology  Cross-Sectional Studies  Metabolic Syndrome X  Research Design | *Gallo, L. C., Penedo, F. J., Carnethon, M., Isasi, C. R., Sotres-Alvarez, D., Malcarne, V. L., et al. (2014). The Hispanic Community Health Study/Study of Latinos Sociocultural Ancillary Study: sample, design, and procedures. *Ethn Dis, 24*(1), 77-83. |
|  | Cardiovascular Diseases/ethnology/psychology  Comorbidity  Diabetes Mellitus/ethnology  Female  Health Status Indicators | *Gallo, L. C., Roesch, S. C., Fortmann, A. L., Carnethon, M. R., Penedo, F. J., Perreira, K., et al. (2014). Associations of chronic stress burden, perceived stress, and traumatic stress with cardiovascular disease prevalence and risk factors in the Hispanic Community Health Study/Study of Latinos Sociocultural Ancillary Study. *Psychosom Med, 76*(6), 468-475. |
|  | Mexican Americans/statistics & numerical data  Occupational Diseases/epidemiology/ethnology | *Gany, F., Novo, P., Dobslaw, R., & Leng, J. (2014). Urban occupational health in the Mexican and Latino/Latina immigrant population: a literature review. *J Immigr Minor Health, 16*(5), 846-855. |
|  | Family/ ethnology/psychology  Health Behavior/ ethnology  Health Status  Mental Health | Katiria Perez, G., & Cruess, D. (2014). The impact of familism on physical and mental health among Hispanics in the United States. *Health Psychol Rev, 8*(1), 95-127. |
|  | Adolescent Development  Environment  Family/ ethnology | *Lawton, K. E., & Gerdes, A. C. (2014). Acculturation and Latino adolescent mental health: integration of individual, environmental, and family influences. *Clin Child Fam Psychol Rev, 17*(4), 385-398. |
|  | Community Health Workers  Diabetes Mellitus, Type 2/ therapy  Hemoglobin A, Glycosylated/metabolism | *Little, T. V., Wang, M. L., Castro, E. M., Jimenez, J., & Rosal, M. C. (2014). Community health worker interventions for Latinos with type 2 diabetes: a systematic review of randomized controlled trials. *Curr Diab Rep, 14*(12), 558. |
|  | Cardiovascular Diseases/ ethnology/ prevention & control  Cohort Studies  Health Behavior/ethnology  Health Services Accessibility | *Schneiderman, N., Chirinos, D. A., Aviles-Santa, M. L., & Heiss, G. (2014). Challenges in preventing heart disease in hispanics: early lessons learned from the Hispanic Community Health Study/Study of Latinos (HCHS/SOL). *Prog Cardiovasc Dis, 57*(3), 253-261. |
|  | Antidepressive Agents/administration & dosage  Anxiety/ethnology  Cardiovascular Diseases/ethnology  Cross-Sectional Studies | *Wassertheil-Smoller, S., Arredondo, E. M., Cai, J., Castaneda, S. F., Choca, J. P., Gallo, L. C., et al. (2014). Depression, anxiety, antidepressant use, and cardiovascular disease among Hispanic men and women of different national backgrounds: results from the Hispanic Community Health Study/Study of Latinos. *Ann Epidemiol, 24*(11), 822-830. |
| 2015 (10) |  |  |
|  | acculturation; ethnic discrimination; regional differences; within group differences | *Arellano-Morales, L., Roesch, S. C., Gallo, L. C., Emory, K. T., Molina, K. M., Gonzalez, P., et al. (2015). Prevalence and Correlates of Perceived Ethnic Discrimination in the Hispanic Community Health Study/Study of Latinos Sociocultural Ancillary Study. *J Lat Psychol, 3*(3), 160-176. |
|  | Metabolic Syndrome X/blood/diagnosis/ethnology  Middle Aged  Obesity/blood/diagnosis/ethnology  Residence Characteristics | *Arguelles, W., Llabre, M. M., Sacco, R. L., Penedo, F. J., Carnethon, M., Gallo, L. C., et al. (2015). Characterization of metabolic syndrome among diverse Hispanics/Latinos living in the United States: Latent class analysis from the Hispanic Community Health Study/Study of Latinos (HCHS/SOL). *Int J Cardiol, 184*, 373-379. |
|  | Cause of Death  Disease/ ethnology  Health Services/ utilization | *Dominguez, K., Penman-Aguilar, A., Chang, M. H., Moonesinghe, R., Castellanos, T., Rodriguez-Lainz, A., & Schieber, R. (2015). Vital signs: leading causes of death, prevalence of diseases and risk factors, and use of health services among Hispanics in the United States - 2009-2013. *MMWR Morb Mortal Wkly Rep, 64*(17), 469-478. |
|  | Cognition/physiology  Neuropsychological Tests | *Gonzalez, H. M., Tarraf, W., Gouskova, N., Gallo, L. C., Penedo, F. J., Davis, S. M., et al. (2015). Neurocognitive function among middle-aged and older Hispanic/Latinos: results from the Hispanic Community Health Study/Study of Latinos. *Arch Clin Neuropsychol, 30*(1), 68-77. |
|  | Diabetes Mellitus, Type 2/epidemiology  Insulin Resistance  Non-alcoholic Fatty Liver Disease  Obesity | *Lazo, M., Bilal, U., & Perez-Escamilla, R. (2015). Epidemiology of NAFLD and Type 2 Diabetes: Health Disparities Among Persons of Hispanic Origin. *Curr Diab Rep, 15*(12), 116. |
|  | Educational Status  Emigrants and Immigrants/psychology  Emotional Adjustment | Lui, P. P. (2015). Intergenerational cultural conflict, mental health, and educational outcomes among Asian and Latino/a Americans: Qualitative and meta-analytic review. *Psychol Bull, 141*(2), 404-446. |
|  | Delivery of Health Care/organization & administration/utilization  Health Policy/legislation & jurisprudence | *Ortega, A. N., Rodriguez, H. P., & Vargas Bustamante, A. (2015). Policy dilemmas in Latino health care and implementation of the Affordable Care Act. *Annu Rev Public Health, 36*, 525-544. |
|  | Community Health Services  Cross-Sectional Studies  Depression/epidemiology  Educational Status  Sleep | *Patel, S. R., Sotres-Alvarez, D., Castaneda, S. F., Dudley, K. A., Gallo, L. C., Hernandez, R., et al. (2015). Social and Health Correlates of Sleep Duration in a US Hispanic Population: Results from the Hispanic Community Health Study/Study of Latinos. *Sleep, 38*(10), 1515-1522. |
|  | Family Relations/psychology  Health Surveys  Metabolic Syndrome X/ethnology/psychology | *Penedo, F. J., Brintz, C. E., MM, L. L., Arguelles, W., Isasi, C. R., Arredondo, E. M., et al. (2015). Family Environment and the Metabolic Syndrome: Results from the Hispanic Community Health Study/Study of Latinos (HCHS/SOL) Sociocultural Ancillary Study (SCAS). *Ann Behav Med, 49*(6), 793-801. |
|  | Digital Divide  Health Literacy  Information Seeking Behavior | Powe, B. D. (2015). Health Information Seeking Among Rural African Americans, Caucasians, and Hispanics: It Is Built, Did They Come? *Nurs Clin North Am, 50*(3), 531-543. |
| 2016 (8) |  |  |
|  | Genetic association study  Anxiety | Dunn, E. C., Sofer, T., Gallo, L. C., Gogarten, S. M., Kerr, K. F., Chen, C. Y., et al. (2016). Genome-wide association study of generalized anxiety symptoms in the Hispanic Community Health Study/Study of Latinos. *Am J Med Genet B Neuropsychiatr Genet*. |
|  | social media; social networking; Hispanic Americans; public health; health behavior | *Hudnut-Beumler, J., Po'e, E., & Barkin, S. (2016). The Use of Social Media for Health Promotion in Hispanic Populations: A Scoping Systematic Review. *JMIR Public Health Surveill, 2*(2), e32. |
|  | Health disparities  Self-rated health  Acculturation | *Lommel, L. L., & Chen, J. L. (2016). The Relationship Between Self-Rated Health and Acculturation in Hispanic and Asian Adult Immigrants: A Systematic Review. *J Immigr Minor Health, 18*(2), 468-478. |
|  | HIV  Pregnancy  STI  Sexual health, Sexual initiation, Sexual risk | Morales-Aleman, M. M., & Scarinci, I. C. (2016). Correlates and predictors of sexual health among adolescent Latinas in the United States: A systematic review of the literature, 2004-2015. *Prev Med, 87*, 183-193. |
|  | CVD; Latina; cancer; health care; women | *Paz, K., & Massey, K. P. (2016). Health Disparity among Latina Women: Comparison with Non-Latina Women. *Clin Med Insights Womens Health, 9*(Suppl 1), 71-74. |
|  | epidemiology; neurocognitive; sleep | Ramos, A. R., Tarraf, W., Daviglus, M., Davis, S., Gallo, L. C., Mossavar-Rahmani, Y, et al. (2016). Sleep Duration and Neurocognitive Function in the Hispanic Community Health Study/Study of Latinos. *Sleep*. |
|  | Occupational health  Organization of work  Immigrant health  Women’s health | *Rodriguez, G., Trejo, G., Schiemann, E., Quandt, S. A., Daniel, S. S., Sandberg, J. C., & Arcury, T. A. (2016). Latina Workers in North Carolina: Work Organization, Domestic Responsibilities, Health, and Family Life. *J Immigr Minor Health, 18*(3), 687-696. |
|  | Allostatic load; Hispanic ethnicity; age patterns; nativity; physiological dysregulation | Salazar, C. R., Strizich, G., Seeman, T. E., Isasi, C. R., Gallo, L. C., Aviles-Santa, M., et al. (2016). Nativity differences in allostatic load by age, sex, and Hispanic background from the Hispanic Community Health Study/Study of Latinos. *SSM Popul Health, 2*, 416-424. |
